# The Relationship of Orthodontic Treatment Need with Periodontal Status, Dental Caries, and Sociodemographic Factors

**DOI:** 10.1100/2012/498012

**Published:** 2012-10-23

**Authors:** Ruhi Nalcaci, Serhat Demirer, Firat Ozturk, Burcu A. Altan, Oral Sokucu, Vildan Bostanci

**Affiliations:** ^1^Department of Orthodontics, Faculty of Dentistry, Suleyman Demirel University, 32260 Isparta, Turkey; ^2^Department of Periodontics, Faculty of Dentistry, Kırıkkale University, 71000 Kırıkkale, Turkey; ^3^Department of Orthodontics, Faculty of Dentistry, İnönü University, 44280 Malatya, Turkey; ^4^Department of Orthodontics, Faculty of Dentistry, Kocaeli University, 41190 Kocaeli, Turkey; ^5^Department of Orthodontics, Faculty of Dentistry, Gaziantep University, 27310 Gaziantep, Turkey; ^6^Department of Periodontics, Faculty of Dentistry, Cumhuriyet University, 58140 Sivas, Turkey

## Abstract

The aim of this study was to determine the relationship of orthodontic malocclusion with periodontal status, dental caries, and sociodemographic status. Our study population consisted of a sample of 836 school children (384 male and 452 female, aged 11–14 years). Four experienced orthodontists and two experienced periodontists performed the clinical examinations. The Treatment Priority Index (TPI), Community Periodontal Index of Treatment Needs (CPITN), decayed, missing, filled teeth (DMFT) scores, and a questionnaire that surveyed socio-demographic status of students were used. Spearman's rank correlation coefficients were used to measure the association between variables. TPI scores showed that 36.4% of the students had normal occlusion, while 41.2% had slight, 15.7% had definite, 4% had severe, and 2.7% had very severe malocclusion. TPI values did not show any significant differences between pupils in different age, gender, socioeconomic status groups, and CPITN scores, whereas there was a significant relationship between TPI and DMFT scores. The orthodontic treatment need was not significantly correlated with CPITN or socio-demographic status; however, the correlation coefficient showed a significant relationship between TPI and DMFT scores.

## 1. Introduction

 Malocclusion is defined as an irregularity of the teeth or an incorrect placement of the dental arches that is outside the ideal range. Besides this irregularity of the teeth or jaws, malocclusion may cause periodontal problems [1], disturbances of oral function such as mastication, swallowing, and speech [2], and psychosocial problems related to impaired dentofacial aesthetics [3].

 Malocclusion is one of the most common dental problems [1, 4]. Over the last three decades there has been a general increase in people's preoccupation with personal aesthetics and their awareness of malocclusion, which has led to a notable increase in the demand for orthodontic treatment [5]. Given the fact that orthodontic treatments are time consuming and expensive, detailed information on the prevalence and distribution of malocclusions is crucial for planning orthodontic treatment within a public health system [6].

 The importance of public health financial management has been increased due to the recent global economic crisis. Hence, governments have been forced to reduce their expenditure on all budget items, including health care. This is especially important in countries where many patients rely on government subsidies to meet their orthodontic treatment needs. Therefore, it is crucial to identify treatment priority among individuals. 

Since the 1950s, several indices have been developed to help obtain quantitative information about the distribution of malocclusions and to record their prevalence and severity [7]. Of these, the most popular indexes have been Summers' Occlusal Index [8], the Treatment Priority Index (TPI) [9], the Handicapping Malocclusion Assessment Record [10], the Need for Orthodontic Treatment Index [11], and the Index of Orthodontic Treatment Need [12]. Using these indices, several studies have presented epidemiological reports of the prevalence of malocclusions in different ethnic groups worldwide. However, in the literature there are limited studies that analyze the relationship between malocclusion and dental problems such as caries and periodontal diseases. The studies that investigated the probable association between malocclusion and various oral hygiene measures revealed inconsistent outcomes [1]; Helm and Petersen [13] and Gábris et al. [14] demonstrated a positive association between malocclusion and periodontal health. However, Katz [15], Buckley [16], and Mtaya et al. [1] found no association between oral hygenie conditions and various orthodontic treatment need. 

 The aims of this study were to survey the relationships between orthodontic and periodontal treatment need, dental caries, and sociodemographic status. These relationships have not been previously studied in the literature with objective measuring scales.

## 2. Methods

 The study population consisted of 836 (384 male and 452 female) school children between 11 and 14 years of age in Sivas, Turkey. The power analysis showed that 836 students were sufficient for our study (*α* = 0,01; *β* = 0,20 (1-*β*) = 0,80; power = 0,8003).

**Figure 1:**
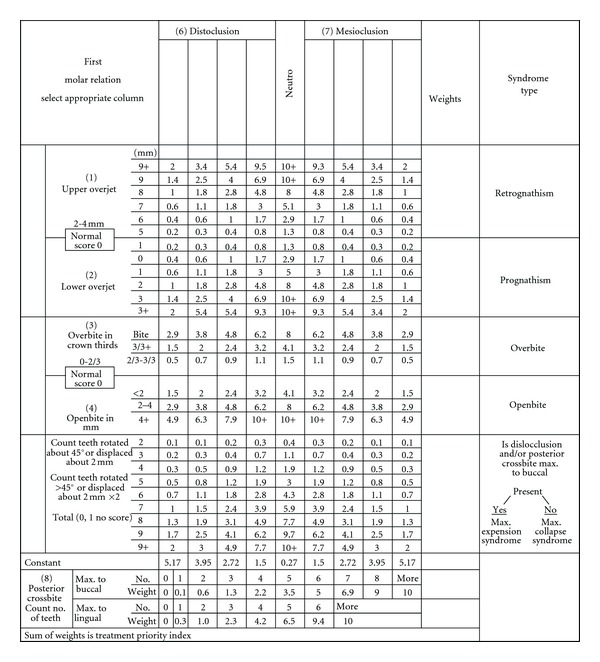
Treatment Index (TPI).

 To determine the socioeconomic condition of the students, a questionnaire was used to survey parents' monthly income and educational status. Treatment Priority Index (TPI) scores were used to determine the severity of malocclusion (Figure 1). To assess periodontal status, the Community Periodontal Index of Treatment Needs (CPITN) was used. Four experienced orthodontists and two experienced periodontists performed the clinical examinations. All of the examiners were trained in the standard use of TPI and CPITN scores before examinations. Subjects were examined with the use of a dental mirror, probe, and Community Periodontal Index probe (for measuring overjet, overbite, open bite, and dental irregularity [17]), under artificial light. 

 The horizontal and vertical incisor relationship, tooth displacement, and occlusion of the buccal segments were measured with TPI. Malocclusions were weighted according to the position of molars in the sagittal plane (Figure 1). A constant value, which also corresponded to the molar occlusion, was added to the TPI score. For each student, recorded malocclusions were summed and a total TPI score was calculated. The severity of malocclusion was assessed according to the Malocclusion Severity Estimate (MSE) [9]. According to the scale modification proposed by Ghafari et al. [18], the constant value for neutrocclusion on the TPI form was scored as normal occlusion. Here, the normal occlusion level was assessed as 0.27 and a score of 0.27–3.99 was regarded as a minor manifestation of malocclusion. In the current study, this modification was preferred. 

 The periodontal status was recorded using the CPITN scores as described by WHO [17]. The CPITN scores were set so that 0 = healthy, 1 = bleeding on gentle probing, 2 = calculus or other plaque-retentive factors, 3 = shallow pocketing of 4-5 mm, and 4 = deep pockets of 6 mm or more. For the periodontal examination we used a dental mirror, an explorer, and the periodontal probe, as recommended by WHO [17]. 

 During oral examination of each child, the number of decayed, missing, or filled teeth was recorded as the DMFT score.

### 2.1. Statistical Analyses

 The data were analyzed using SPSS for Windows, version 13.0 (SPSS Inc., Chicago, IL, USA). TPI measurements for the different genders were compared using Student's *t* tests and age groups were compared using analysis of variance (ANOVA). The effects of age, gender, mothers' and fathers' education levels, parents' monthly income, and CPITN scores on TPI scores were examined using the chi-square test. Spearman's rank correlation coefficients were estimated to provide a measure of the association between TPI, CPITN, and DMFT scores. Levels of statistical significance were set at *P* < 0.05. 

## 3. Results

 The children's parents' monthly income and educational status are shown in Tables 1 and 2. In the present study, the TPI scores showed that 36.4% of the students had normal occlusion, while 41.2% had slight, 15.7% had definite, 4% had severe, and 2.7% had very severe malocclusion (Table 3).

TPI values did not show any significant differences between pupils in different age, gender, and socioeconomic status groups, as calculated based on the children's mothers' and fathers' education and monthly income (Table 4). 

According to the CPITN scores, 36.6% of students had a healthy periodontium, 35.3% showed bleeding on gentle probing, and 21.9% had signs calculus or other plaque-retentive factors. Only 13 students (1.5%) had shallow pocketing of 4-5 mm and 39 students (4.6%) had deep pockets of 6 mm or more. These scores were rated according to periodontal treatment need, as described by WHO. As such, 309 students (36.6%) had no need for periodontal treatment (TN0), 298 students (35.3%) needed only oral hygiene instruction (TN1), 23.4% of the students were assessed as TN2, while 39 students (4.6%) were in TN3, with the greatest need for treatment. 

TPI scores did not show any significant differences with CPITN scores (*X* ^2^ = 19.22, *P* = 0.257, *P* > 0.05). In Table 5, detailed data showing the comparison of TPI scores with CPITN scores are provided. 

The correlation coefficients between TPI, CPITN, and DMFT scores are shown in Table 6. No significant relationship was found between TPI-CPITN scores (*r* = 0.043, *P* = 0.211). Correlation coefficients between TPI and DMFT scores, on the other hand, showed a significant positive relationship (*r* = 0.98, *P* = 0.004) as TPI scores increased in higher DMFT scores. 

The correlation coefficients for the relationship between CPITN-DMFT scores were also significant (*r* = 0.210, *P* = 0.001). CPITN scores increased with increase of DMFT scores.

## 4. Discussion

 Information obtained from cross-sectional studies is crucial for many reasons, such as monitoring trends in oral health, evaluating levels of dental need, assessing the effectiveness of oral-health promotional strategies, planning oral-health policies, and emphasizing dental issues politically [19–21]. To the best of our knowledge, this study is the first cross-sectional study of Turkish school children that analyzes the relationship between malocclusion and periodontal status, dental problems, and sociodemographic status. In the wider literature the vast majority of studies investigated malocclusion and orthodontic treatment need from the orthodontic perspective only.

In this study, paediatric orthodontic treatment need was evaluated using the TPI. This index has been shown to be a good epidemiological indicator of malocclusion [18]. In a 1993 review, Tang and Wei [22] maintained that the TPI is a practical method that requires less chair time compared with the Occlusal Index. These authors also stated that since malocclusion is a multifaceted problem, there is no universally accepted index that defines all the characteristics of malocclusion. 

In our study, TPI values did not show any significant correlation with age, gender, parental education, or monthly income. We also showed that 63.6% of our study population exhibited from slight to very severe malocclusion. In a previous study conducted in Turkey, Güray et al. [23] found 72.3% of 483 students required orthodontic treatment in a low socioeconomic standard primary school in the Konya district. Similarly in 1998, Ugur et al. evaluated 572 children of high socioeconomic status and found that 59.6% needed orthodontic treatment [24]. 

In 1999, Tickle et al. [25] investigated the relationship between socioeconomic status and both normatively assessed and self-perceived need for orthodontic treatment. Their results showed that there was a predominance of deprived children in the group with the highest normatively measured orthodontic treatment need. The authors concluded that socioeconomic status did affect normatively measured orthodontic treatment need, though the mechanism was unclear. In 2009, Mtaya et al. [1] studied the prevalence of malocclusion and its association with sociodemographic characteristics, caries, and level of oral hygiene in 12 to 14 year-old schoolchildren residing in two socioeconomically different districts of Tanzania and found a significant increase in the occurrence of open bite in the group of deprived children. In contrast to these studies, the results of our study show a high need of orthodontic treatment, regardless of socioeconomic status. 

The results of our statistical analysis demonstrated that TPI scores did not show a significant correlation with CPITN scores. This result was consistent with studies by Katz [15] and Buckley [16]. Similar to our results, these authors found no association between the amount of plaque, calculus, gingivitis, or pocketing with the prevalence of malocclusion. However, Helm and Petersen [13], Gábris et al. [14], and Mtaya et al. [1] showed a correlation between malocclusion and periodontal health. Orthodontic malocclusion is believed to be an important factor in the aetiology of periodontal disease. Maintenance of a healthy dentition with aligned teeth in their arches was considered anatomically and functionally critical, as irregular teeth may increase retention sites and lead to periodontal problems. However, CPITN measures periodontal treatment need of the entire jaw, thus local periodontal problems may be masked by healthy areas. 

Due to our results, TPI and DMFT scores were positively correlated, showing that malocclusions were associated with decayed, missed, or filed teeth, as expected. Our results were similar with previous studies. Mtaya et al. [1] found that children with experience of caries (DMFT > 0) were almost two times more likely to have any type of malocclusion compared with their counterparts without caries (DMFT = 0). Furthermore, Stahl and Grabowski [26] reported dental caries and premature loss of primary teeth as predisposing factors for occlusal and space anomalies in the mixed and permanent dentition. These authors also stated that students with DMFT > 0 were two times more likely than their peers without caries to be diagnosed with a midline shift. Some of the authors explained the relationship of malocclusion and dental caries by the incidence of untreated proximal caries in primary molars or early loss of a second primary molar leading to forward drift of the first permanent molar, ultimately leading to change in the molar relationship [27, 28].

In Turkey, 75% of the cost of orthodontic treatment is covered by the public dental services for children up to the age of 18 years, regardless of normative orthodontic treatment need. This leads to overcrowded clinics and delay of treatment for those with very severe malocclusion. After the worldwide financial crisis, like most other countries, Turkey experienced a reformation of healthcare policies. This new situation calls for measures such as treatment priority indices for planning appropriate orthodontic services and allocating of limited funds according to treatment priority. In our opinion, we need more than TPI classification in order to assess the individual treatment need from different perspectives, including dental care and oral health awareness. With more detailed information we can select patients most likely to benefit from orthodontic treatment, preventing unnecessary waste of public resources and minimising the occurrence of adverse outcomes.

## 5. Conclusion

 Although we did not find a relationship between orthodontic treatment need and periodontal treatment need, we find a positive correlation with TPI and DMFT scores. Thus, preventing the children from dental caries will decrease future orthodontic treatment need. Also we should address oral health awareness and children must be encouraged to attend their dentist regularly. 

## Figures and Tables

**Table 1:** Parental monthly income.

Parents monthly income	*n*	%
200 € and less	97	11.6
200 €–400 €	210	25.1
400 €–650 €	278	33.2
650 €–1000 €	200	23.9
1000 € and more	51	6.2

Total	836	100.0

**Table 2:** Parental educational status.

Educational status	Mother		Father	
	*n*	%	*n*	%
Elementary school	406	48.5	159	19
Middle school	163	19.5	159	19
High school	191	23	325	38.8
University	76	9	193	23.2

Total	836	100	860	100

**Table 3:** Orthodontic treatment need according to TPI scores.

Treatment need	*n*	%
Normal occlusion	305	36.4
Slight malocclusion	344	41.2
Definite malocclusion	132	15.7
Severe malocclusion	33	4
Very severe malocclusion	22	2.7

Total	836	100.0

**Table 4:** Comparison of TPI scores with age, gender, parental education, and parental monthly income.

	Age		Gender		Mothers' education		Fathers' education		Parents' monthly income	
	*x* ^2^	*P*	*x* ^2^	*P*	*x* ^2^	*P*	*x* ^2^	*P*	*x* ^2^	*P*
TPI	11.29	0.50	4.83	0.30	12.29	0.72	10.80	0.82	13.30	0.65

**Table 5:** Comparison of TPI and CPITN scores.

TPI cassification	Periodontal treatment need				Total
	TN(0)	TN(I)	TN(II)	TN(III)
Normal occlusion					
*n*	112	112	70	11	305
%	36.8%	36.8%	22.8%	3.6%	100%
Slight malocclusion					
*n*	135	113	77	19	344
%	39.3%	32.9%	22.3%	5.5%	100.0%
Definite malocclusion					
*n*	47	45	34	6	132
%	35.8%	34.3%	25.3%	4.5%	100.0%
Severe malocclusion					
*n*	5	18	9	1	33
%	14.3%	54.3%	28.6%	2.9%	100.0%
Very severe malocclusion					
*n*	7	6	7	2	22
%	31.8%	27.3%	31.8%	9.1%	100.0%

Total					
*n*	306	294	197	39	836
%	36.6%	35.3%	23.4%	4.6%	100.0%

*X* ^2^ = 19.22, *P* = 0.257, *P* > 0.05.

**Table 6:** Correlation coefficients between CPITN, TPI, and DMFT scores.

	CPITN		TPI		DMFT	
	*r*	*P*	*r*	*P*	*r*	*P*
CPITN	—		0.043	0.211	0.210	0.001*
TPI	0.043	0.211	—		0.098	0.004*
DMFT	0.210	0.001*	0.98	0.004*	—	

*P < 0.05.

## References

[1] Mtaya M, Brudvik P, Åstrøm AN (2009). Prevalence of malocclusion and its relationship with socio-demographic factors, dental caries, and oral hygiene in 12- to 14-year-old Tanzanian schoolchildren. *European Journal of Orthodontics*.

[2] Proffit WR, Fields HW (2007). *Contemporary Orthodontics*.

[3] Kenealy P, Frude N, Shaw W (1989). An evaluation of the psychological and social effects of malocclusion: some implications for dental policy making. *Social Science and Medicine*.

[4] Dhar V, Jain A, Van Dyke TE, Kohli A (2007). Prevalence of gingival diseases, malocclusion and fluorosis in school-going children of rural areas in Udaipur district. *Journal of Indian Society of Pedodontics and Preventive Dentistry*.

[5] Perillo L, Masucci C, Ferro F, Apicella D, Baccetti T (2010). Prevalence of orthodontic treatment need in southern Italian schoolchildren. *European Journal of Orthodontics*.

[6] Foster TD, Menezes DM (1976). The assessment of occlusal features for public health planning purposes. *American Journal of Orthodontics*.

[7] Massler M, Frankel JM (1951). Prevalence of malocclusion in children aged 14 to 18 years. *American Journal of Orthodontics*.

[8] Summers CJ (1971). The occlusal index: a system for identifying and scoring occlusal disorders. *American Journal of Orthodontics*.

[9] Grainger RM (1967). *Orthodontic Treatment Priority Index*.

[10] Salzmann JA (1968). Handicapping malocclusion assessment to establish treatment priority. *American Journal of Orthodontics*.

[11] Espeland LV, Ivarsson K, Stenvik A (1992). A new Norwegian index of orthodontic treatment need related to orthodontic concern among 11-year-olds and their parents. *Community Dentistry and Oral Epidemiology*.

[12] Brook PH, Shaw WC (1989). The development of an index of orthodontic treatment priority. *European Journal of Orthodontics*.

[13] Helm S, Petersen PE (1989). Causal relation between malocclusion and caries. *Acta Odontologica Scandinavica*.

[14] Gábris K, Márton S, Madléna M (2006). Prevalence of malocclusions in Hungarian adolescents. *European Journal of Orthodontics*.

[15] Katz RV (1978). An epidemiologic study of the relationship between various states of occlusion and the pathological conditions of dental caries and periodontal disease. *Journal of Dental Research*.

[16] Buckley LA (1980). The relationships between irregular teeth, plaque, calculus and gingival disease. A study of 300 subjects. *British Dental Journal*.

[17] World Health Organization. Geneva (1997). *Oral Health Surveys-Basic Methods*.

[18] Ghafari J, Locke SA, Bentley JM (1989). Longitudinal evaluation of the Treatment Priority Index (TPI). *American Journal of Orthodontics and Dentofacial Orthopedics*.

[19] Burt BA (1997). How useful are cross-sectional data from surveys of dental caries?. *Community Dentistry and Oral Epidemiology*.

[20] Jamieson LM, Thomson M (2002). Dental health, dental neglect, and use of services in an adult Dunedin population sample. *New Zealand Dental Journal*.

[21] Jamieson LM, Thomson WM (2002). The Dental Neglect and Dental Indifference scales compared. *Community Dentistry and Oral Epidemiology*.

[22] Tang ELK, Wei SHY (1993). Recording and measuring malocclusion: a review of the literature. *American Journal of Orthodontics and Dentofacial Orthopedics*.

[23] Güray E, Orhan M, Ertas E, Doruk C (1994). Konya Yöresi İlkokul Çocuklarında Treatment Priority Index (TPI) Uygulaması (Epidemiyolojik Çalışma). *Türk Ortodonti Dergisi*.

[24] Uğur T, Ciğer S, Aksoy A, Telli A (1998). An epidemiological survey using the Treatment Priority Index (TPI). *European Journal of Orthodontics*.

[25] Tickle M, Kay EJ, Beam D (1999). Socio-economic status and orthodontic treatment need. *Community Dentistry and Oral Epidemiology*.

[26] Stahl F, Grabowski R (2004). Malocclusion and caries prevalence: is there a connection in the primary and mixed dentitions?. *Clinical Oral Investigations*.

[27] Koch G, Poulsen S (2001). *Pediatric Dentistry: A Clinical Approach*.

[28] Mitchell L (2007). *An Introduction to Orthodontics*.

